# Foot-and-mouth disease virus infection suppresses autophagy and NF-*к*B antiviral responses via degradation of ATG5-ATG12 by 3C^pro^

**DOI:** 10.1038/cddis.2016.489

**Published:** 2017-01-19

**Authors:** Xuxu Fan, Shichong Han, Dan Yan, Yuan Gao, Yanquan Wei, Xiangtao Liu, Ying Liao, Huichen Guo, Shiqi Sun

**Affiliations:** 1 State Key Laboratory of Veterinary Etiological Biology and National Foot and Mouth Disease Reference Laboratory, Lanzhou Veterinary Research Institute, Chinese Academy of Agricultural Sciences, Lanzhou, Gansu, P. R. China; 2 Key Laboratory of Zoonosis of Ministry of Agriculture, College of Veterinary Medicine, China Agricultural University, Haidian District, Beijing, P. R. China; 3 Department of Avian Diseases, Shanghai Veterinary Research Institute, Chinese Academy of Agricultural Sciences, Shanghai, P. R. China

## Abstract

Autophagy-related protein ATG5-ATG12 is an essential complex for the autophagophore elongation in autophagy, which has been reported to be involved in foot-and-mouth disease virus (FMDV) replication. Previous reports show that ATG5-ATG12 positively or negatively regulates type I interferon (IFN-*α*/*β*) pathway during virus infection. In this study, we found that FMDV infection rapidly induced LC3 lipidation and GFP-LC3 subcellular redistribution at the early infection stage in PK-15 cells. Along with infection time course to 2–5 h.p.i., the levels of LC3II and ATG5-ATG12 were gradually reduced. Further study showed that ATG5-ATG12 was degraded by viral protein 3C^pro^, demonstrating that FMDV suppresses autophagy along with viral protein production. Depletion of ATG5-ATG12 by siRNA knock down significantly increased the FMDV yields, whereas overexpression of ATG5-ATG12 had the opposite effects, suggesting that degradation of ATG5-ATG12 benefits virus growth. Further experiment showed that overexpression of ATG5-ATG12 positively regulated NF-*к*B pathway during FMDV infection, marked with promotion of IKK*α*/*β* phosphorylation and I*κ*B*α* degradation, inhibition of p65 degradation, and facilitation of p65 nuclear translocation. Meanwhile, ATG5-ATG12 also promoted the phosphorylation of TBK1 and activation of IRF3 via preventing TRAF3 degradation. The positive regulation of NF-*к*B and IRF3 pathway by ATG5-ATG12 resulted in enhanced expression of IFN-*β*, chemokines/cytokines, and IFN stimulated genes, including anti-viral protein PKR. Altogether, above findings suggest that ATG5-ATG12 positively regulate anti-viral NF-*κ*B and IRF3 signaling during FMDV infection, thereby limiting FMDV proliferation. FMDV has evolved mechanisms to counteract the antiviral function of ATG5-ATG12, via degradation of them by viral protein 3C^pro^.

Autophagy is not only a conserved dynamic cellular process to degrade cellular damaged organelles and long-lived proteins but also an evolutional pathway to degrade intracellular pathogens including bacterium and viruses.^1, 2, 3, 4^ Autophagy is processed by the formation of double membrane autophagosome, the fusion of autophagosome with lysosome to form autolysosome, and the digestion of contents of the autolysosome.^5^ It has been reported that almost 31 kinds of ATGs participate in the mammalian autophagy.^6^ Among them, two Beclin 1 complex (hVPS34-Beclin1-mATG14 and hVPS34-Beclin 1-UVRAG) and two ubiquitin-like conjugation systems (ATG5-ATG12-ATG16L and LC3 conjugation systems) are essential in nucleation, expansion of the autophagosomal membrane, and fusion of lysosome with autophagosome.^7, 8^ Autophagy is regarded as one of the several autonomous arms of intrinsic innate immunity, which helps host cells to defend viral infection.^1, 9^ Viruses will manipulate autophagy during their infection, mainly target to ATGs and Beclin1, to counteract the antiviral effect.^10^ It has been reported that N-terminal caspase-recruitment domains (CARDs) of ATG5-ATG12 associate with retinoic acid-inducible gene I (RIG-I) and IFN-*β* promoter stimulator-1(IPS-1) (also called MAVS, Cardif, and VISA),^11^ negatively regulating the production of type I IFN and cytokines.^12, 13^ Therefore, ATGs are involved in innate immune signaling in virus-specific and cell type-dependent manner.

Foot-and-mouth disease virus (FMDV) is a positive single-stranded RNA virus belonging to *Picornaviridae*. It causes a highly contagious viral disease called foot-and-mouth disease (FMD) in cloven-hoofed animals.^14, 15, 16^ The double stranded viral RNAs (dsRNAs) produced during viral genome replication are mainly recognized by melanoma-differentiation-associated gene 5 (MDA5). The signals are then transmitted to IPS-1 via the interaction of CARDs, eventually activating the transcription factor NF-*κ*B and IRF3, and triggering the production of the anti-viral IFNs and inflammatory cytokines/chemokines.^17, 18, 19^ FMDV has developed sophisticated strategies to antagonize the host antiviral responses: viral leader proteinase (L^pro^) has been identified as an IFN-*β* antagonist, via degradation of NF-*κ*B subunit p65,^20^ decrease of IRF3/7 expression,^21^ and inhibition of RIG-I, TBK1, TRAF3/6 ubiquination;^22^ viral proteinase 3C^pro^ antagonizes innate immune signaling via cleavage of NEMO,^23^ or blockage of STAT1/STAT2 nuclear translocation.^24^ Other FMDV proteins, such as 2B, 2C, 3A and VP3, also participate in negative regulation of the type I IFN pathway.^25, 26, 27, 28^

Studies on other members of *Picornaviridae,* including poliovirus, enterovirus type 71(EV71), and coxsackie virus have shown that these viruses hijack autophagy to facilitate their replication.^29, 30, 31, 32^ The role of autophagy on manipulation of FMDV production has not got a unanimous conclusion yet. One group shows that autophagy may facilitate FMDV growth as FMDV yields are reduced in Atg5^−/−^ MEF cells.^33^ However, other researchers have reported that FMDV utilizes autophagy to promote viral replication.^33, 34, 35^ Here, we reported that FMDV infection rapidly induced autophagy at the early infection stage, and subsequently suppressed autophagy via degradation of ATG5-ATG12 when viral protease 3C^pro^ was synthesized. Replenishment of ATG5-ATG12 in PK-15 cells upregulated the expression of anti-viral proteins via recovery of NF-*κ*B and IRF3 activation, thereby limiting FMDV replication.

## Results

### Kinetic induction and suppression of autophagy in FMDV-infected PK-15 cells

**Figure 1:**
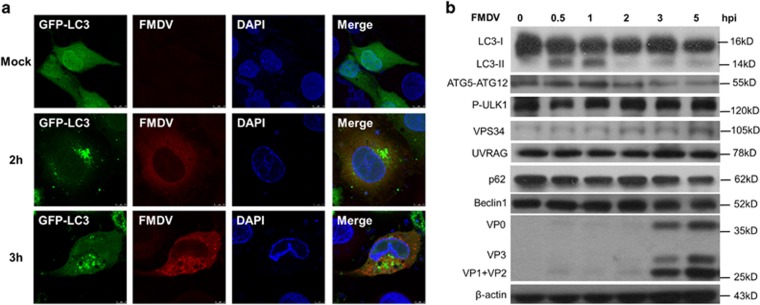
FMDV infection rapidly induces autophagy in PK-15 cells, and the autophagy is subsequently suppressed along with infection time course. (**a**) FMDV infection induces GFP-LC3 puncta. PK-15 cells were transfected with GFP-tagged LC3 expression plasmid, followed by FMDV infection. Cells were then subjected to indirect immunofluorescence at indicated time points, and the signals of PC3-GFP and FMDV proteins were observed under confocal microscope. Green signals represent GFP-LC3, red signals represent FMDV, and nucleus was stained with DAPI. (**b**) Autophagy is rapidly induced after FMDV infection and subsequently suppressed along the infection time course. Cells were infected with FMDV and harvested at the indicated time points, followed by Western blot analysis with the indicated antibodies

We first examined whether FMDV infection promotes the aggregation of GFP-LC3 to autophagosome in PK-15 cells. Cells were transfected with plasmid-encoding GFP-LC3, followed by infection with FMDV serotype Asia I. In agreement with the previous report, GFP-LC3 formed punctate structure at 2 and 3 h.p.i., comparing the uniform distribution in both nuclear and cytosol in mock-infected cells (Figure 1a). This result confirms that FMDV infection induces autophagy in PK-15 cells.

Next, the kinetic protein levels of the autophagy-related proteins were examined. As shown in Figure 1b and Supplementary Figure S1, the level of lipidation from LC3-II was significantly increased as early as 0.5 to 1 h.p.i., and was diminished from 2 to 5 h.p.i. In consistency with the kinetics of LC3-II, the levels of ATG5-ATG12 conjugate were increased from 0.5 to 1 h.p.i., and declined from 2 to 5 h.p.i. However, the protein levels of p-ULK1 and p62, the upstream markers of autophagic mTOR pathway, were decreased from 0.5 to 1 h.p.i. and almost restored the protein level to that before infection from 2 to 5 h.p.i. On the contrary, the protein levels of VPS34, UVRAG, and beclin1, the markers of class III PI3K pathway, were not significantly affected by FMDV infection. These results suggest that FMDV infection rapidly induces autophagy via the mTOR pathway upon entry step, independent of class III PI3K pathway, consistent with previous reports.^33^ The interesting finding is that the FMDV-induced autophagy is suppressed via degradation of ATG5-ATG12 after 2 h.p.i.

### Degradation of ATG5-ATG12 via viral protein 3C^pro^

**Figure 2:**
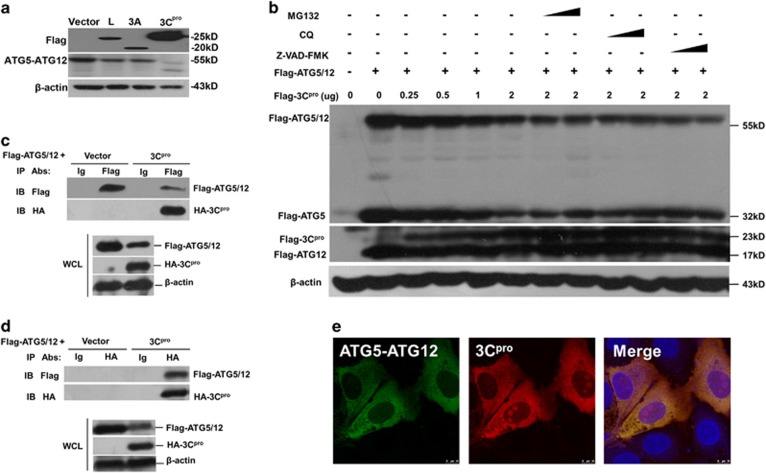
FMDV suppresses autophagy via degradation of ATG5-ATG12 by 3C^pro^. (**a**) FMDV 3C^pro^ is responsible for ATG5-ATG12 degradation. PK-15 cells were transfected with vector, Flag-L, Flag-2A, and Flag-3C plasmids. Western blot analysis was performed using anti-Flag, anti-ATG5-ATG12, and *β*-actin. (**b**) Effect of proteasome inhibitor MG132, lysosome inhibitor CQ, and caspase inhibitor Z-VAD-FMK on 3C^pro^-induced ATG5-ATG12 degradation. PK-15 cells were transfected with Flag-ATG5-ATG12 plasmid and increasing dose of Flag-3C^pro^ plasmid (0, 0.25, 0.5, 1, 2 *μ*g), in the presence or absence of MG132 (2 *μ*M, 20 *μ*M), CQ (50 *μ*M, 100 *μ*M) or Z-VAD-FMK (10 *μ*M, 50 *μ*M). The expression level of Flag-ATG5-ATG12 and Flag-3C^pro^ was detected with Western blot using anti-Flag. (**c** and **d**) 3C^pro^ directly interacts with ATG5-ATG12 and mediates the degradation. PK-15 cells were co-transfected with empty vector and Flag-ATG5-ATG12 plasmid, or HA-3C^pro^ plasmid and Flag-ATG5-ATG12 plasmid. The cells were lysed after 24 h and immunoprecipitated with anti-HA or anti-Flag antibodies, followed by Western blot analysis. (**e**) 3C^pro^ co-localizes and interacts with ATG5-ATG12. PK-15 cells were co-transfected with HA-3C^pro^ or Flag-ATG5-ATG12 expressing plasmid for 24 h. Cells were subjected to immunostaining with anti-Flag (green) and anti-HA (red)

Several reports show that FMDV proteases L^pro^ and 3C^pro^ are responsible for cleavage of many proteins and thereby they manipulate signaling pathways.^20, 21, 22, 23, 24^ Therefore, viral proteins responsible for ATG5-ATG12 degradation were screened via overexpression of Flag-tagged viral protein L^pro^, 3A, and 3C^pro^ in PK-15 cells, respectively. As shown in Figure 2a, the successful expression of viral proteins was easily detected by anti-Flag. The level of ATG5-ATG12 conjugate was slightly diminished in cells expressing either L^pro^ or 3A. Surprisingly, a significant decrease and degradation of ATG5-ATG12 conjugate was observed in the presence of 3C^pro^, demonstrating that viral protease 3C^pro^ is responsible for the degradation of AGT5-ATG12, thereby suppressing the ongoing autophagy.

Next, we examined whether proteasome, lysosome, or caspase were involved in FMDV 3C^pro^-induced AGT5-ATG12 depletion. The proteasome inhibitor MG132, lysosome inhibitor CQ, and caspase inhibitor Z-VAD-FMK were used to evaluate the effects. Results showed that degradation of Flag-ATG5-ATG12 was dose-dependent on Flag-3C^pro^, and none of the inhibitors rescued the degradation (Figure 2b). These results suggest that 3C^pro^-induced depletion of ATG5-ATG12 is independent of proteasome, lysosome, or caspase. Thus, we hypothesize that 3C^pro^ directly binds, cleaves, and degrades ATG5-ATG12. To investigate the interaction between 3C^pro^ and ATG5-ATG12, PK-15 cells were co-transfected with Flag-ATG5, Flag-ATG12, and HA-3C^pro^. Cells co-transfected with Flag-ATG5, Flag-ATG12, and HA-vector were included in a parallel experiment as control. The cell lysates were immune-precipitated with anti-Flag, followed by Western blot analysis. As shown in Figure 2c, anti-Flag successfully pulled down both Flag-ATG5-ATG12 conjugate and HA-3C^pro^, confirming the interaction between Flag-ATG5-ATG12 and HA-3C^pro^. Using anti-HA to pull down HA-3C^pro^, Flag-ATG5-ATG12 was also co-immunoprecipitated with HA-3C^pro^ (Figure 2d), further confirming the interaction between Flag-ATG5-ATG12 and HA-3C^pro^.

Furthermore, immunofluorescence experiment was performed to confirm the interaction of ATG5-ATG12 and 3C^pro^. Perfect co-localization of Flag-ATG5-ATG12 (green) and HA-3C^pro^ (red) was observed under confocal microscope (Figure 2e), with uniform distribution in the cytoplasm. It was noted that a small portion of HA-3C^pro^ entered into nucleus. Collectively, these results demonstrate that 3C^pro^ directly interacts with and degrades ATG5-ATG12.

### Inhibition of FMDV proliferation by replenishment of ATG5-ATG12 in cells

**Figure 3:**
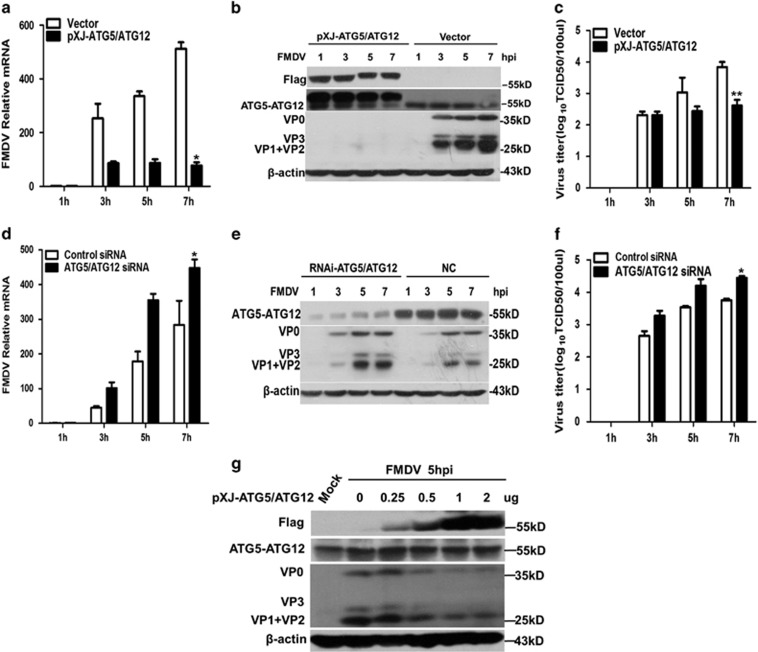
Overexpression of ATG5-ATG12 reduces FMDV yields and knock down of ATG5-ATG12 increases FMDV yields. (**a**-**c**) Overexpression of ATG5-ATG12 suppresses FMDV replication. PK-15 cells were transfected with vector or Flag-ATG5-ATG12 plasmid for 24 h, followed with FMDV infection (MOI=1) for 1, 3, 5, and 7 h. (**a**) Total RNAs were extracted and the levels of viral mRNA were determined with quantitative real-time RT-PCR analysis. (**b**) Protein samples were subjected to Western blot analysis to detect the expression of Flag-ATG5-ATG12 and viral proteins VP0, VP1, VP2, and VP3. (**c**) Supernatants were collected and subjected to TCID_50_ assay to measure virus titer. (**d**-**f**) Knock down of ATG5-ATG12 enhances FMDV yields. PK-15 cells were transfected with control siRNA (NC) or siRNA targeting to ATG5 and ATG12 for 36 h, followed with FMDV infection (MOI=1) for 1, 3, 5, and 7 h. (**d**) Total RNAs were extracted and the levels of viral mRNA were determined with quantitative real-time RT-PCR analysis. (**e**) Protein samples were subjected to Western blot analysis to detect the expression of Flag-ATG5-ATG12 and viral proteins VP0, VP1, VP2, and VP3. (**f**) Supernatants were collected and subjected to TCID_50_ assay to measure virus titer. (**g**) ATG5-ATG12 inhibits viral replication in a dose-dependent manner. PK-15 (1.2 × 10^6^ cells each well) cells were transfected with increasing dose of Flag-ATG5 and Flag-ATG12 plasmid (0, 0.25, 0.5, 1 or 2 *μ*g), followed with FMDV infection (MOI=1) for 5 h. Protein samples were subjected to Western blot analysis to detect the expression of Flag-ATG5-ATG12 and viral proteins VP0, VP1, VP2, and VP3. Above data are representative of three independent experiments. Graphs show mean±S.D.; *n*=3. *P<0.05; **P<0.01

ATG5-ATG12 conjugate has an essential role in both canonical and non-canonical autophagy pathways.^36^ To better understand the role of ATG5-ATG12 conjugate in FMDV infection, Flag-ATG5 and Flag-ATG12 were exogenously co-expressed in PK-15 cells, followed by FMDV infection. As shown in Figures 3a and c, replenishment of ATG5-ATG12 significantly suppressed FMDV replication by reducing the viral mRNA transcription, viral protein translation, and virus particle release. In addition, replenishment of ATG5-ATG12 decreased the FMDV-induced cytopathic effect (CPE) (Supplementary Figure S2A). Furthermore, FMDV replication was suppressed by ATG5-ATG12 in a dose-dependent manner (Figure 3g).

To further confirm above results, the knockdown effects were confirmed by Western blot analysis using anti-ATG5-ATG12 (Figure 3e). Depletion of ATG5 and ATG12 by siRNA knock down significantly enhanced FMDV growth by promoting viral mRNA transcription, viral proteins production, and viral particle release, compared with that in the control siRNA-transfected cells (Figures 3d and f).

To assess whether the inhibitory effect of ATG5-ATG12 on FMDV replication is cell type specific, IBRS-2 cells and BHK-21 cells were used. Results showed that the production of viral proteins was dramatically decreased in IBRS-2 cells by overexpression of ATG5-ATG12 (Supplementary Figure S2B). However, replenishment of ATG5-ATG12 had no inhibition effect on FMDV replication in BHK-21 cells (Supplementary Figure S2C). We speculate that the above difference might be due to type I IFN defective in BHK-21 cells,^37^ which reminds us that the inhibitory effect of ATG5-ATG12 on FMDV proliferation may correlate with type I IFN production. Altogether, above results suggest that ATG5-ATG12 suppresses FMDV infection, probably via regulation of type I IFN production.

### Involvement of ATG5-ATG12 in FMDV-triggered production of IFN-*β* and IFN stimulated genes

**Figure 4:**
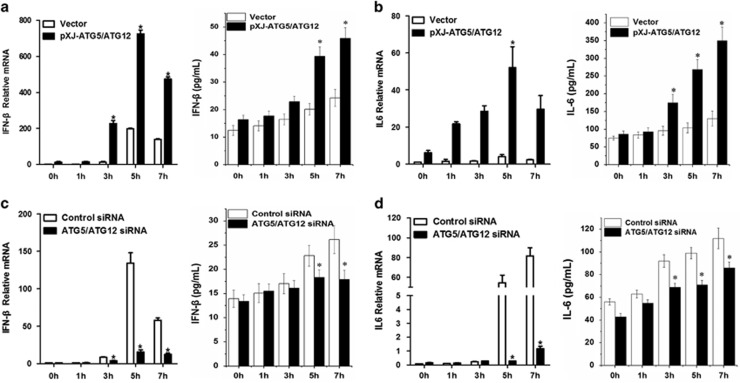
ATG5-ATG12 positively regulates FMDV-induced IFN-*β* and IL-6. (**a**) Overexpression of ATG5-ATG12 enhances IFN-*β* production at mRNA and protein level. PK-15 cells were transfected with vector, Flag-ATG5 and Flag-ATG12 plasmid for 24 h, followed with FMDV infection (MOI=1). Total RNAs were extracted at indicated time points, and IFN-*β* mRNA was quantified with quantitative real-time RT-PCR analysis. The culture medium was collected at indicated time points for quantification of the secretion of IFN-*β* with ELISA. (**b**) Overexpression of ATG5-ATG12 enhances IL-6 production at mRNA level and protein level. The experiments were performed similarly to (**a**). The level of IL-6 mRNA and the secretion of IL-6 were detected with quantitative real-time RT-PCR analysis and ELISA, respectively. (**c**) Knockdown of ATG5-ATG12 reduces IFN-*β* production at mRNA level and protein level. PK-15 cells were transfected with control siRNA (NC) or ATG5-ATG12 siRNA for 36 h, followed with FMDV infection (MOI=1). The level of IFN-*β* mRNA and the secretion of IFN-*β* were detected with quantitative real-time RT-PCR analysis and ELISA, respectively. (**d**) Knockdown of ATG5-ATG12 reduces IL-6 production at mRNA level and protein level. The experiments were similarly performed as (**c**). The level of IL-6 mRNA and the secretion of IL-6 were detected with quantitative real-time RT-PCR analysis and ELISA, respectively. Above data are representative of three independent experiments. Graphs show mean±S.D.; *n*=3. *P<0.05

To investigate the role of ATG5-ATG12 conjugate on FMDV-triggered type I IFN and chemokines/cytokines production, PK-15 cells were co-transfected with Flag-ATG5 and Flag-ATG12, followed by FMDV infection. The mRNA transcription and protein production of IFN-*β* and IL-6 were measured by quantitative real-time RT-PCR and ELISA, respectively. Results revealed that expression of ATG5-ATG12 strongly increased the expression of IFN-*β* and IL-6 at 5–7 h.p.i. (Figures 4a and b), compared with that in the vector-transfected cells. On the contrary, depletion of ATG5-ATG12 by siRNA knock down significantly reduced the expression of IFN-*β* and IL-6 at 5–7 h.p.i., compared with that in the control siRNA-transfected cells (Figures 4c and d). Consistent with the above results, the mRNA levels of chemokines CXCL10, RIG-I, and MDA5 were significantly increased in ATG5-ATG12 overexpressing cells, whereas they decreased in ATG5-ATG12 depletion cells (Supplementary Figures S3A and B). These results confirmed that ATG5-ATG12 suppresses FMDV proliferation via promotion of IFN-*β* and cytokines/chemokines production.

### Positive regulation of NF-*κ*B-signaling pathway by ATG5-ATG12 during FMDV infection

**Figure 5:**
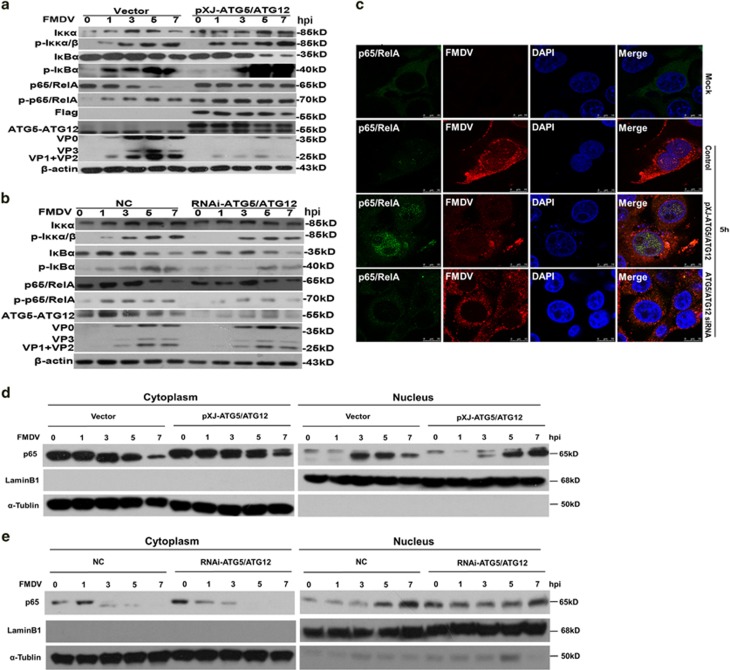
ATG5-ATG12 positively regulates NF-*κ*B p65 signaling during FMDV infection. (**a**) Overexpression of ATG5-ATG12 promotes the NF-*κ*B signaling during FMDV infection. PK-15 cells were transfected with vector, Flag-ATG5 and Flag-ATG12 plasmid for 24 h, followed with FMDV infection (MOI=1). Cells were harvested at indicated time points, and protein samples were prepared. IKK*α*, phospho-KK*α*/*β*,I*κ*B*α*, phospho-I*κ*B*α*, p65, phospho-p65, Flag-ATG5-ATG12, ATG5-ATG12, and viral proteins were detected with Western blot analysis. (**b**) Knock down of ATG5-ATG12 blocks NF-*κ*B signaling during FMDV infection. PK-15 cells were transfected with control siRNA (NC) or ATG5-ATG12 siRNA for 36 h, followed with FMDV infection (MOI=1). Cells were harvested at indicated time points and protein samples were prepared. IKK*α*, phospho-KK*α*/*β*, I*κ*B*α*, phospho-I*κ*B*α*, p65, phospho-p65, Flag-ATG5-ATG12, ATG5-ATG12, and viral proteins were detected with Western blot analysis. (**c**) Overexpression of ATG5-ATG12 promotes p65 nuclear translocation. PK-15 cells were transfected with vector, Flag-ATG5 and Flag-ATG12 plasmid, or ATG5-ATG12 siRNA, respectively. Cells were mock-infected or infected with FMDV for 5 h. Immunostaining was performed using specific antibodies against p65 (green) and FMDV (red). Nucleus was stained with DAPI. (**d**) Overexpression of ATG5-ATG12 promotes p65 nuclear translocation. PK-15 cells were transfected with Flag-ATG5 and Flag-ATG12 plasmid for 24 h, followed with FMDV infection. Cytoplasmic and nuclear extracts were prepared and the levels of p65 were analyzed with Western blot. Lamin B1 was detected as loading control of the nuclear fraction, and *α*-tubulin was detected as loading control of the cytoplasmic fraction. (**e**) Knockdown of ATG5-ATG12 inhibits p65 nuclear translocation. PK-15 cells were transfected with control siRNA (NC) or ATG5-ATG12 siRNA for 36 h, followed with FMDV infection. Cytoplasmic and nuclear extracts were prepared, and the levels of p65 were analyzed with Western blot. Lamin B1 was detected as loading control of the nuclear fraction, and *α*-tubulin was detected as loading control of the cytoplasmic fraction. Above experiments were performed in triplicates

To investigate the role of ATG5-ATG12 in the regulation of NF-*κ*B pathway, Flag-ATG5 and Flag-ATG12 were expressed in PK-15 cells, followed by FMDV stimulation. The levels of phosphor-IKK*α*/*β*, phosphor-I*κ*B*α*, total I*κ*B*α*, NF-*κ*B subunit p65, and phosphor-p65 were examined using specific antibodies. As shown in Figure 5a, in vector-transfected cells, efficient virus replication was detected with anti-FMDV, and the endogenous ATG5-ATG12 conjugate was detected as single band. The levels of phosphor-IKK*α*/*β* and phosphor-I*κ*B*α* were moderately increased at 1 and 3 h.p.i., and accumulated at 5 and 7 h.p.i. (Figure 5a, Supplementary Figures S4A and B), respectively. Consistent with the increased IKK*α*/*β* and I*κ*B*α* phosphorylation, the levels of total I*κ*B*α* slightly decreased at 5 and 7 h.p.i. (Figure 5a and Supplementary Figure S4B). As a consequence, p65/RelA was phosphorylated from 1 to 7 h.p.i. (Figure 5a and Supplementary Figure S4C). Consistent with the previous study,^20^ total p65/RelA was gradually decreased from 3 to 7 h.p.i. (Figure 5a and Supplementary Figure S4C), confirming the degradation of p65/RelA. These results show that FMDV infection slightly activates the upstream signaling of p65/RelA, and inhibits the NF-*к*B signaling by degradation of p65/RelA. In Flag-ATG5-ATG12 expressing cells, virus replication was suppressed, marked as the minimum level of viral proteins detected (Figure 5a). Endogenous ATG5-ATG12 conjugate and exogenous Flag-ATG5-ATG12 conjugate were detected as two bands with anti-ATG5-ATG12 (Figure 5a). A bit more phosphor-IKK*α*/*β* was detected at 7 h.p.i., and significant increase of phosphor-I*κ*B*α* and degradation of total I*κ*B*α* was detected at 5 and 7 h.p.i., compared with that in vector-transfected cells (Figure 5a, Supplementary Figures S4A and B). Surprisingly, more phosphor-p65/RelA was detected at each time point, and the degradation of p65/RelA was prevented (Figure 5a and Supplementary Figure S4C). Above results suggest that FMDV infection blocks the activation of NF-*κ*B via p65/RelA degradation; however, replenishment of ATG5-ATG12 strengthens NF-*к*B signaling via promotion of IKK*α*/*β* phosphorylation, I*κ*B*α* degradation, p65/RelA phosphorylation, and prevention of p65/RelA degradation.

In ATG-5-ATG12 knock down cells, the phosphor-IKK*α*/*β* and phosphor-I*к*B was less than that in control cells from 1 to 7 h.p.i., and I*к*B degradation was comparable in both cells at 5 and 7 h.p.i. (Figure 5b, Supplementary Figures S4D and E). Less phosphor-p65/RelA was detected from 1 to 7 h.p.i., and p65/RelA degradation was comparable in both cells at 5 and 7 h.p.i. (Figure 5b and Supplementary Figure S4F). Again, a bit more viral proteins VP0, VP1, and VP3 were detected in ATG5-ATG12 knock down cells than that in control cells (Figure 5b).

Next, indirect immunofluorescence experiment was performed to compare the nuclear translocation of p65/Rel in mock-infected cells, vector-transfected cells (followed by FMDV infection), Flag-ATG5-ATG12 expressing cells (followed by FMDV infection), and ATG5-ATG12 knock down cells (followed by FMDV infection). As shown in Figure 5c, p65/RelA was uniformly distributed in cytosol in mock-infected cells. In FMDV-infected cells, the signal of p65/RelA was weak, due to virus-induced degradation, and a small portion of p65/RelA entered into the nucleus at 5 h.p.i. (Figure 5c). As expected, in ATG5-ATG12 overexpressing cells, nuclear signals of p65/RelA were intensified, and the signals of FMDV proteins were diminished (Figure 5c). In ATG5-ATG12 knock down cells, almost no nuclear signals of p65/RelA were observed, and the signals of viral proteins were stronger than that in ATG5-ATG12 overexpressing cells (Figure 5c). As demonstrated above, NF-*к*B activity was dramatically enhanced in the presence of ATG5-ATG12 during FMDV infection, thereby suppressing virus replication.

We further verified the above results by nuclear/cytosol fractionation of p65/RelA. As shown in Figure 5d, in vector-transfected cells, nuclear p65/RelA was increased at 3 h after FMDV infection, and gradually diminished from 5 to 7 h.p.i., due to the degradation of total p65/RelA. In ATG5-ATG12 overexpressing cells, the degradation of total p65/RelA was prevented, nuclear p65/RelA was increased at 3 h.p.i., and higher level of nuclear p65/RelA was detected at 5 and 7 h.p.i., compared with that in vector-transfected cells (Figure 5d, Supplementary Figures S5A and B). In ATG5-ATG12 knock down cells, both cytoplasmic p65/RelA and nuclear p65/RelA was less than that in control cells at 7 h.p.i. (Figure 5e, Supplementary Figures S5C and D). Altogether, all these findings demonstrate that ATG5-ATG12 inhibits FMDV-induced p65/RelA degradation and promotes its nuclear translocation, thereby promoting antiviral immune responses and suppressing FMDV proliferation.

### Positive regulation of IRF-3 signaling pathway during FMDV infection by ATG5-ATG12

**Figure 6:**
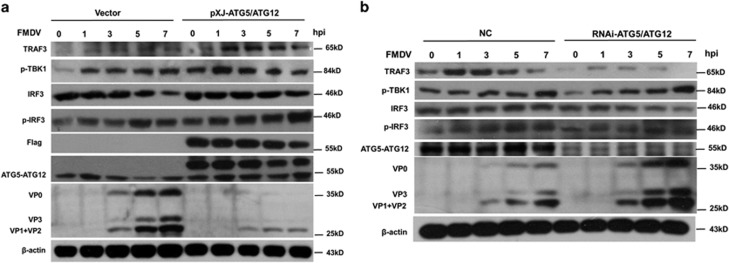
ATG5-ATG12 positively regulates IRF3 signaling during FMDV infection. (**a**) Overexpression of ATG5-ATG12 promotes the IRF3 signaling during FMDV infection. PK-15 cells were transfected with vector, Flag-ATG5, and Flag-ATG12 plasmid for 24 h, followed with FMDV infection. Cells were harvested at 0, 1, 3, 5, and 7 h.p.i. The levels of TRAF3, phospho-TBK1, IRF3, phospho-IRF3, Flag-ATG5-ATG12, ATG5-ATG12, and viral proteins were analyzed with Western blot. (**b**) Knockdown of ATG5-ATG12 reduces the IRF3 signaling during FMDV infection. PK-15 cells were transfected with control siRNA (NC), or ATG5-ATG12 siRNA for 36 h, followed with FMDV infection. Cells were harvested at 0, 1, 3, 5, and 7 h.p.i. The levels of TRAF3, phospho-TBK1, IRF3, phospho-IRF3, ATG5-ATG12, and viral proteins were analyzed with Western blot. Above experiments were performed in triplicates

To investigate the role of ATG5-ATG12 on IRF3 signaling, ATG5 and ATG12 were co-expressed in PK-15 cells, followed by FMDV infection. Results showed that in transfected cells, TRAF3 slightly increased at the early stage of FMDV infection and decreased subsequently (Figure 6a and Supplementary Figure S6A). TBK1 and IRF3 were phosphorylated, along the time course of infection (Figure 6a and Supplementary Figure S6B-D). It was noted that total IRF3 was decreased at 7 h.p.i., probably due to virus infection-induced degradation or phosphorylation and nuclear translocation of IRF3. However, in cells overexpressing Flag-ATG5-ATG12, the level of TRAF3 was markedly increased, and more phosphor-TBK1 was detected after FMDV infection, than that in vector-transfected cells. More phosphor-IRF3 was detected, and the degradation of IRF3 was prevented, in the presence of Flag-ATG5 and Flag-ATG12 (Figure 6a and Supplementary Figure S6A-D). Above results were further confirmed by knock down experiment. As shown in Figure 6b and Supplementary Figures S6E-H, depletion of ATG5-ATG12 promoted the degradation of TRAF3, and the levels of the phosphor-TBK1 and phosphor-IRF3 were lower than that in control cells. Altogether, these results demonstrate that ATG5-ATG12 positively regulates IRF3-mediated activation of I-IFN signal pathway.

### The role of PKR in ATG5-ATG12-mediated antiviral response

**Figure 7:**
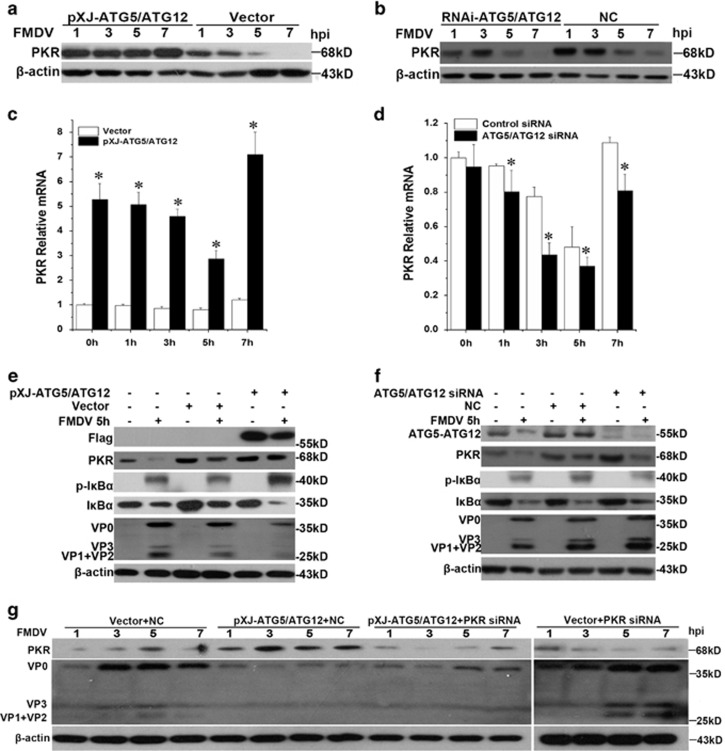
PKR has an important role in ATG5-ATG12 mediated antiviral responses. (**a**) Overexpression of ATG5-ATG12 prevents the degradation of PKR during FMDV infection. PK-15 cells were transfected with vector, Flag-ATG5, and Flag-ATG12 plasmid for 24 h, followed with FMDV infection. Cell lysates were prepared at 1, 3, 5, and 7 h.p.i., and the levels of PKR were examined with Western blot using anti-PKR. (**b**) Knock down of ATG5-ATG12 reduces PKR protein level. PK-15 cells were transfected with control siRNA or ATG5-ATG12 siRNA for 36 h, followed with FMDV infection. Cell lysates were prepared at 1, 3, 5, 7 h.p.i., and the levels of PKR protein were examined with Western blot using anti-PKR. (**c**) Overexpression of ATG5-ATG12 dramatically increases PKR mRNA transcription. The experiments were similarly performed as (**a**), and the PKR mRNA was quantified using quantitative real-time RT-PCR. The results represent the means and standard deviations of data from three independent experiments. *P<0.05. (**d**) Knock down of ATG5-ATG12 moderately inhibits PKR mRNA transcription. The experiments were similarly performed as (**b**), and the PKR mRNA was quantified using quantitative real-time RT-PCR. The results represent the means and standard deviations of data from three independent experiments. *P<0.05. (**e**) ATG5-ATG12 triggers the upregulation of PKR and increases the phosphorylation level of I*κ*B. PK-15 cells were transfected with vector, Flag-ATG5, and Flag-ATG12 plasmid for 24 h, followed with FMDV infection. Cell lysates were prepared at 5 h.p.i., and the levels of Flag-ATG5-ATG12, PKR, phospho-I*к*B*α*, I*к*B*α*, and viral proteins were examined with Western blot. (**f**) Knock down of ATG5-ATG12 reduces PKR protein expression and phosphorylation level of I*κ*B. PK-15 cells were transfected with control siRNA or ATG5-ATG12 siRNA for 36 h, followed with FMDV infection. Cell lysates were prepared at 5 h.p.i., and the levels of ATG5-ATG12, PKR, phospho-I*к*B*α*, I*к*B*α*, and viral proteins were examined with Western blot. (**g**) PKR has an important role in anti-viral effect of ATG5-ATG12. PK-15 cells were co-transfected with vector and control siRNA (NC), Flag-ATG5/Flag-ATG12 and control siRNA (NC), Flag-ATG5/Flag-ATG12 and PKR siRNA, or vector and PKR siRNA, followed with FMDV infection. The cells harvested at 1, 3, 5, and 7 h.p.i., and the levels of PKR and viral proteins were analyzed with Western blot

We found that FMDV infection reduced the level of PKR protein at 5 and 7 h.p.i. in vector or control siRNA transfected cells (Figures 7a and b). However, overexpression of ATG5-ATG12 prevented the degradation of PKR and upregulated the expression of PKR at 7 h.p.i. (Figure 7a). Knockdown of ATG5-ATG12 accelerated the degradation of PKR at 5 and 7 h.p.i. (Figure 7b). In addition, quantitative real-time RT-PCR experiments further confirmed that overexpression of ATG5-ATG12 dramatically enhanced PKR transcription, compared with the control groups (Figure 7c). In contrast, depletion of ATG5-ATG12 by siRNA knock down had opposite effects (Figure 7d). Furthermore, along with the increased level of PKR in cells overexpressing Flag-ATG5-ATG12, the level of phosphor-I*κ*B was increased and degradation of I*κ*B was enhanced during FMDV infection (Figure 7e and Supplementary Figure S7A). However, knock down of ATG5-ATG12 had the opposite effects on PKR expression and I*κ*B phosphorylation and degradation (Figure 7f and Supplementary Figure S7B). These results suggest that ATG5-ATG12 upregulates the expression of PKR, positively regulating NF-*κ*B signaling via phosphorylation of I*κ*B.

To confirm the antiviral role of PKR in cells replenishing with ATG5-ATG12, PKR was knocked down by siRNA in cells overexpressing Flag-ATG5-ATG12 or vector-transfected cells, followed by FMDV infection. Western blot analysis showed that depletion of PKR resulted in production of more viral proteins, compared with that in control siRNA transfected cells (Figure 7g). These results demonstrate that PKR has an important anti-viral role, acting as an ATG5-ATG12 stimulated gene.

## Discussion

**Figure 8:**
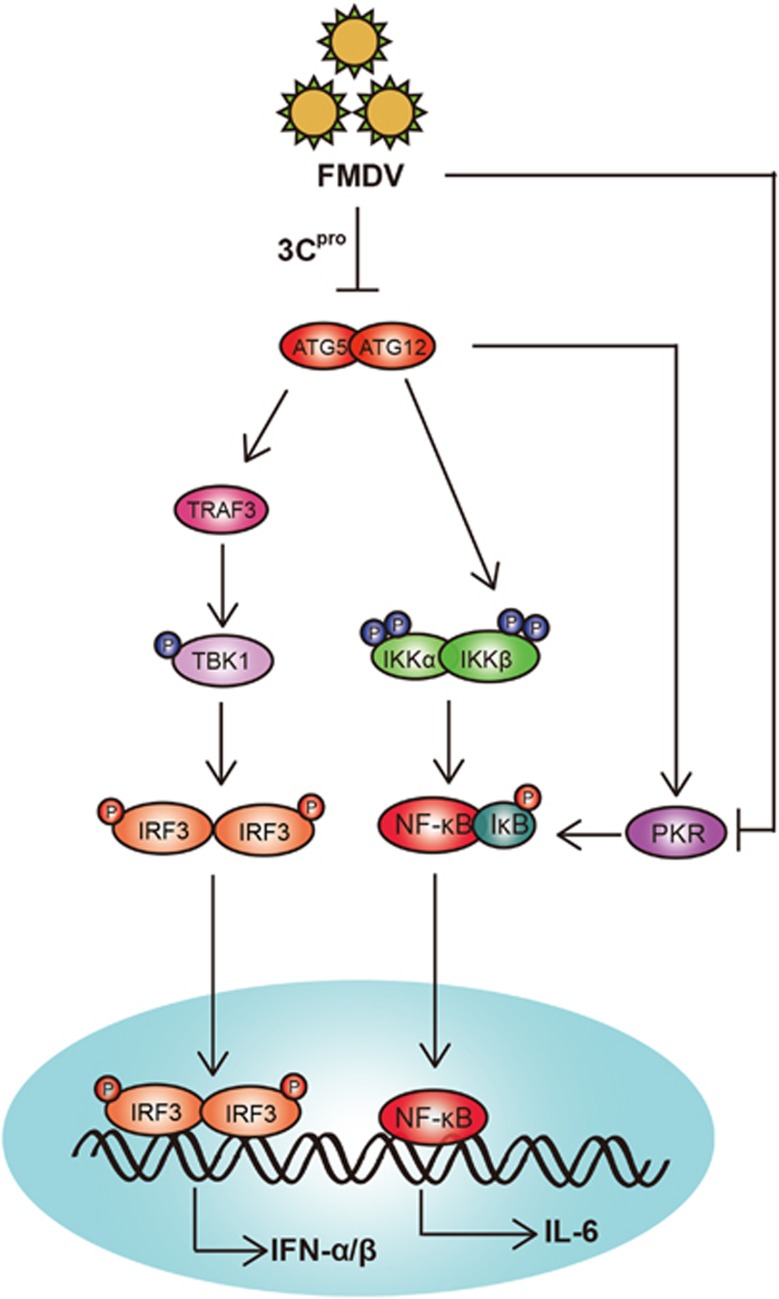
Model of ATG5-ATG12 involvement in FMDV-induced type IIFN signal pathway. FMDV infection rapidly induces autophagy. Subsequently, the autophagy is probably suppressed via degradation of ATG5 and ATG12 via FMDV 3C^pro^. Replenishment of ATG5-ATG12 conjugate promotes the phosphorylation of IKK*α*/*β*, phosphorylation and degradation of I*κ*B*α*, subsequently promoting the nuclear translocation of p65. Meanwhile, ATG5-ATG12 increases the IRF3 activity through stabilizing TRAF3 and increasing the phosphorylation of TBK1 and IRF3. Moreover, ATG5-ATG12 also can block the FMDV-trigged PKR reduction, resulting in increased p-IkB and the subsequent activation of NF-*κ*B. Then the activation of NF-*κ*B and IRF upregulates the transcription of kB-dependent genes and type I IFN

Autophagy is a double-edged sword that either helps host cell eliminate the pathogen or is hijacked by invasive viruses to facilitate their own proliferation.^38, 39^ Genetic knockout studies have suggested an important role of ATGs in the protection of mice, human, worms, and slime molds against viral or protozoal pathogens.^11, 13, 40^ In this study, we first report that ATG5-ATG12 have an anti-viral role during FMDV (Asia I/Jiangsu/China/2005) infection, the brief regulation was diagrammatized in Figure 8. We find that FMDV infection induces autophagy at early stage of infection in PK-15 cells, which is marked by LC3 lipidation and LC3-GFP punctate structure distribution in cells. It suggested that FMDV promotes the autophagy in the early stage in order to help its infection. However, FMDV suppresses the autophagy via degradation of ATG5-ATG12 proteins by viral protease 3C^pro^. The antiviral role of autophagy in our study is contradictory to the two previous reports, where silencing of LC3 or ATG dramatically decreases FMDV yields.^33, 34^ This contradiction may be due to various virus strains or cell types used in these studies.

It showed that p-ULK1 and p62 are gradually decreased with the FMDV infection. Although ULK1 level seems to increase after 2 h.p.i, the total degree of ULK1 is not increased as compared with original state at 0 h.p.i. ULK1 activity would be modulated by mTORC1 and involved in autophagy induction.^41^ It is needed in early steps of autophagosome biogenesis and will be degraded by the lysosome in the subsequent autophagesome. Similarly, p62 protein serves as a link between LC3 and ubiquitinated substrates.^42^ The LC3II brings the p62 to aggregate into the autophagesome, which was degraded by lysosome and leads to the decrease of p62.^43, 44^ To summarize, we conclude that the activation autophagy could be due to FMDV-cell surface receptor binding, and the suppression of autophagy after 2-3 h.p.i. could be attributed to the expression of viral protease 3C^pro^.

It has been reported that ATG5-ATG12 conjugate interacts directly with the IPS-1 and RIG-I through the CARDs, resulting in inhibition of type I IFN production under physiological conditions and may have an important role in maintaining cellular homeostasis. Thus, in our study, we demonstrate that ATG5-ATG12 promotes NF-*к*B activity during FMDV infection, by promotion of the phosphorylation of IKK*α*/*β* and degradation of I*к*B, and prevention of p65 degradation. Meanwhile, ATG5-ATG12 increases the IRF3 activity through stabilization of TRAF3, thereby increasing the phosphorylation of TBK1 and IRF3.

It has been reported that p65 protein and IRF3/7 protein are degraded by L^pro^, and NEMO protein and KPNA1 protein are degraded by 3C^pro^ during FMDV infection, resulting in the inhibition of the innate immune responses.^20, 21, 22, 23^ In this study, FMDV has evolved mechanisms to degrade ATG5-ATG12 by 3C^pro^, counteracting the antiviral ATG5-ATG12. Thus, the FMDV-encoded proteases have an important role to escape the innate immune responses. Here, we also found that FMDV escape the antiviral responses by depletion of antiviral protein PKR, and the expression of ATG5-ATG12 recovers the expression and accumulation of PKR. The depletion mechanisms of PKR need further investigation.

In conclusion, we have shown that FMDV suppresses NF-*к*B and IRF3 by degradation of ATG5-ATG12 via 3C^pro^, thereby leading to evade the innate immune response. ATG5-ATG12 could be used as a target for drug design to combat FMDV infection. Unfortunately, the mechanism of prevention of p65 degradation by the replenishment of ATG5-ATG12 is unclear. Further analysis would be required to understand the regulation mechanisms of ATG5-ATG12 in viral replication and antiviral responses.

## Materials and methods

### Cells and viruses

Porcine kidney cells, PK-15 cells, IBRS-2 cells, and baby hamster kidney cells (BHK-21) were obtained from ATCC. All cell lines are maintained in Dulbecco's modified Eagle's medium (DMEM, Gibco, Carlsbad, CA, USA) supplemented with 10% fetal bovine serum (FBS, Sigma, Louis, MO, USA), 100 *μ*g/ml penicillin, and 100 *μ*g/ml streptomycin (Invitrogen, Carlsbad, CA, USA) in a humidified incubator with 5% CO_2_ at 37 °C.

FMDV (Asia I/Jiangsu/China/2005) (GenBank Accession No. EF149009) was stored at OIE/National Foot-and-Mouth Disease Reference Laboratory (Lanzhou, Gansu, P.R. China). It was propagated in BHK-21 cells. The virus titer was determined by Tissue Culture Infective Dose (TCID_50_) assay on BHK-21 cells.

### Antibodies, siRNAs, and inhibitor

Anti-ATG5-ATG12 mouse monoclonal antibody (A2859), anti-LC3II rabbit polyclonal antibody (L7543), anti-VPS34 rabbit polyclonal antibody (V9764), anti-UVRAG rabbit polyclonal antibody (U7508), anti-Flag rabbit polyclonal antibody (F7425), and the secondary antibodies conjugating with HRP, FITC, or TRITC were purchased from Sigma-Aldrich (Louis, MO, USA). Anti-IKK*α* rabbit polyclonal antibody (sc-7607), anti-I*κ*B*α* rabbit polyclonal antibody (sc-371), anti-PKR mouse monoclonal antibody (sc-6284), anti-Lambin B1 mouse monoclonal antibody (sc-377000), anti-*α*-Tubulin mouse monoclonal antibody (sc-398103), anti-*β*-actin mouse monoclonal antibody (sc-47778), and anti-p65 mouse monoclonal antibody (sc-8008) were purchased from Santa Cruz Biotechnology (Santa Cruz, CA, USA). Anti-phospho-I*κ*B*α* mouse monoclonal antibody (#9246), anti-phospho-IKK*α* (Ser176)/IKK*β* (Ser177) rabbit monoclonal antibody (#2078), anti-phospho-TBK1 (Ser172) rabbit monoclonal antibody (#5483), anti-phospho-IRF-3 (Ser396) rabbit monoclonal antibody (#4947), anti-TRAF3 rabbit polyclonal antibody (#4729), and anti-IRF-3 rabbit monoclonal antibody (#4302) were purchased from Cell Signal Technology (CST, Beverly, MA, USA). Anti-phospho-ULK1 (S556) rabbit monoclonal antibody (ab133747), Anti-SQSTM1/p62 mouse monoclonal antibody (ab56416), anti-ATG16L1 rabbit monoclonal antibody (ab187671), and anti-Beclin1 rabbit polyclonal antibody (ab62557) were purchased from Abcam (Cambridge, UK). Polyclonal pig antiserum against FMDV was provided by OIE reference laboratory of China (Lanzhou, Gansu, P.R. China).

Special siRNAs targeting ATG5 (sc-41446), ATG12 (sc-72579), or PKR (sc-36264) were purchased from Santa Cruz Biotechnology, Inc (Santa Cruz, CA, USA). The proteasome inhibitor MG132 was purchased from Merck & Co (Darmstadt, Germany). The caspase inhibitor benzyloxycarbony (Cbz)-l-Val-Ala-Asp (OMe)-fluoromethylketone (Z-VAD-FMK) and the lysosome inhibitor chloroquine diphosphate (CQ) were purchased from Sigma-Aldrich.

### Western blot analysis

Protein samples were subjected to SDS-PAGE, transferred to a poly vinylidene difluoride membrane (Merck Millipore, MA, USA), and incubated with indicated antibody. The protein signals were visualized using the Supersignal West Pico chemiluminescence ECL kit (Pierce, MA, USA).

### Construction of plasmids

The porcine ATG5 and ATG12 genes were prepared by RT-PCR using total RNA extracts from PK-15 cells. ATG5 gene was amplified using two primers: forward primer 5′-GCGGATCCACAGATGACAAAGATGTGCTTC-3′ and reverse primer 5′-TAGGTACCTCAGTCTGTTGGCTGGGGCA-3′. ATG5 gene was then cloned into pXJ40-Flag vector between the restriction enzyme sites of *BamH*I and *Kpn*I (pXJ40-Flag-ATG5). ATG12 gene was amplified using two primers: forward primer 5′-ATAAGCTTGCAGAGGAGCCGGAGTCT-3′ and reverse primer 5′-TAGGTACCTCATCCCCAAGCCTGAGATT-3′. ATG12 gene was then cloned into pXJ40-Flag vector between the restriction enzyme sites of *Hind*III and *Kpn*I (pXJ40-Flag-ATG12). Both genes were fused with Flag tag at N-terminus. The two recombinant plasmids were verified using standard sequencing techniques. The plasmids pEGFP-LC3, pXJ40-Flag-L, pXJ40-Flag-3A, and pXJ40-Flag-3C were constructed by our lab.

### Cell transfection

Cells (approximately 1 × 10^5^) were seeded on six-well plates at 80% confluence. On the second day, cells were transfected with indicated plasmids (3 *μ*g/well) using Lipofectamine 2000 according to the manufacturer's manual (Invitrogen). Cells were then infected with 1 MOI of FMDV at 24 h post transfection, harvested at indicated time points post infection, and subjected to Western blot analysis, immunofluorescence, and quantitative real-time RT-PCR.

### RNA interference

Cells were plated in six-well plates at 50% confluence. On the second day, cells were subjected to siRNA transfection (40–80 pmol/well) using Lipofectamine RNAi MAX according to the manufacturer's manual (Invitrogen). All siRNAs used in this study were purchased from Santa Cruz Biotechnology, Inc (Santa Cruz, CA, USA). Cells were incubated for 36 h and exposed to FMDV infection at 1 MOI. Cells were harvested at indicated time points and subjected to Western blot analysis, immunofluorescence, real-time RT-PCR.

### Quantitative real-time RT-PCR

Total RNA was extracted using TRIzol reagent according to the manufacturer's manual (Invitrogen). RNA was reverse transcribed into cDNA using an oligo (dT) primer and reverse transcriptase M-MLV (Takara, Dalian, China). cDNA was then subjected to real-time PCR quantification using SYBR green PCR master mix (Takara, Dalian, China). For detection of specific genes, the following primers were used: IFN-*β* forward primer 5′-GCTAACAAGTGCATCCTCCAAA-3′ and IFN-*β* reverse primer 5′-AGCACATCATAGCTCATGGAAAGA-3′ IL-6 forward primer 5′-CTGCTTCTGGTGATGGCTACTG-3′ and IL-6 reverse primer 5′-GGCATCACCTTTGGCATCTT-3′ CXCL10 forward primer 5′-ATGGTTCATCATCCCGAGCT-3′ and CXCL10 reverse primer 5′-CCAGGACTTGGCACATTCACTAA-3′ RIG-I forward primer 5′-CTTGCAAGAGGAATACCACTTAAACCCAGAGAC and reverse primer TTCTGCCACGTCCAGTCAATATGCCAGGTTT; MDA5 forward primer 5′-TCTGCTTATCGCTACCACAGTGGCAGA and reverse primer 5′ TGCTCTCATCAGCTCTGGCTCGACC; FMDV forward primer 5′-TTCGGCCTTTGATGCTAACCACTG-3′ and FMDV reverse primer 5′-GCATCCCGCCCTCAACAACAAT-3′. For mRNA quantification, the house keeping gene GAPDH was used as a reference point using the following primers: GAPDH forward primer 5′-ACATGGCCTCCAAGGAGTAAGA-3′ and GAPDH reverse primer 5′-GATCGAGTTGGGGCTGTGACT-3′.

The levels of respective mRNA transcript in each sample were assayed three times and normalized to that of GAPDH mRNA. Relative transcript levels were quantified by the 2^−△△CT^ (where CT is threshold cycle) method and were shown as fold change relative to the level of the mock-treated control cells.

### Co-immunoprecipitation

The co-immunoprecipitation analysis was performed using Pierce co-immunoprecipitation kit (26149) (Pierce, MA, USA). PK-15 cells were transfected with the appropriate plasmids and harvested at 24 h post transfection. Target proteins were immunoprecipitated according to the manufacturer's protocol.

### Immunofluorescence

Cells grown on glass slides were washed with PBS, fixed in 4% paraformaldehyde for 20 min, and permeabilized with 0.2% Triton X-100 for 15 min. Cells were then incubated in 5% newborn calf serum in PBS at 37 °C for 1 h, followed by incubation with primary antibody and secondary antibody for 2 h, respectively. After being washed with PBS for three times, cells were further incubated with DAPI (Beyotime, Jiangsu, China) for staining of nucleus. After being washed with PBS twice, cells were analyzed using laser-scanning confocal microscope (LSCM, Leica SP8, Solms, Germany) at the wavelengths of 405 nm, 488 nm, and 561 nm.

### TCID_50_ assay

Briefly, PK-15 cells were seeded in 96-well plate at 90% confluence, serial 10-fold dilutions of virus prepared in FBS-free DMEM were added in 50 *μ*l volume to each well, and plates were incubated at 37 °C for 72 h. Each of the samples was monitored for the presence or absence of CPE. TCID_50_ was calculated by Reed–Muench method.

### ELISA

The levels of IFN-*β* and IL-6 in the supernatant were determined using porcine IFN-*β* QuantiKine ELISA kit (R&D Systems, MN, USA) and porcine IL-6 QuantiKine ELISA kit (Novatein Biosciences, Woburn, MA, USA) according to the manufacturer's instructions.

### Nuclear/cytosol fractionation assay

The cells were collected and subjected to nuclear and cytosol fractionation using Nuclear/Cytosol Fractionation Kit (BioVision, Milpitas, CA, USA), following the protocols recommended by the manufacturer.

### Statistics analysis

The results were representative of at least three independent experiments and were presented as the mean±standard deviation (S.D.) of triplicate experiments. Statistics were performed using the unpaired student *t*-test. The statistical significance was set at *P*<0.05 (*) and *P*<0.01 (**).
