# Supplementary material for: Foot-and-mouth disease virus infection suppresses autophagy and NF-кB antiviral responses via degradation of ATG5-ATG12 by 3Cpro
Source: Cell Death Dis. 2017 Jan 19;8(1):e2561–. doi: 10.1038/cddis.2016.489 (PMC5386389; doi:10.1038/cddis.2016.489)
Supplement: Supplementary Information [file cddis2016489x1.docx]

**Foot-and–mouth disease virus infection suppresses autophagy and NF-кB antiviral responses via degradation of ATG5-ATG12 by 3C^pro^**

Xuxu Fan^1†^, Shichong Han^1,3†^, Dan Yan^1^, Yuan Gao^1^, Yanquan Wei^1^, Xiangtao Liu^1^, Ying Liao^2*^, Huichen Guo^1*^, Shiqi Sun^1*^

^1^State Key Laboratory of Veterinary Etiological Biology and National Foot and Mouth Disease Reference Laboratory, Lanzhou Veterinary Research Institute, Chinese Academy of Agricultural Sciences, Xujiaping 1, Lanzhou, Gansu, 730046, P. R. China

^2^Department of Avian Diseases, Shanghai Veterinary Research Institute, Chinese Academy of Agricultural Sciences, Ziyue Road 518, Shanghai 200241, P. R. China

^3^Key Laboratory of Zoonosis of Ministry of Agriculture, College of Veterinary Medicine, China Agricultural University, No. 2,Yuan MingYuan West Road, Haidian District, Beijing 100193, P. R. China

**^*^**Correspondence author:

State Key Laboratory of Veterinary Etiological Biology and National Foot and Mouth Disease Reference Laboratory, Lanzhou Veterinary Research Institute, Chinese Academy of Agricultural Sciences, Xujiaping 1, Lanzhou, Gansu, 730046, The People’s Republic of China

Tel: 86-0931-8312213; Fax: 86-0931-8340977;

E-mail: [sunshiqi@caas.cn, guohuichen](mailto:sunshiqi@caas.cn,%20guohuichen)@caas.cn, [liaoying@shvri.ac.cn](mailto:liaoying@shvri.ac.cn)

^†^These authors contributed equally to this work and should be considered co-first authors

**Running title: Foot-and–mouth disease virus degrades ATG5-ATG12 and suppresses** NF-кB **antiviral responses**

**
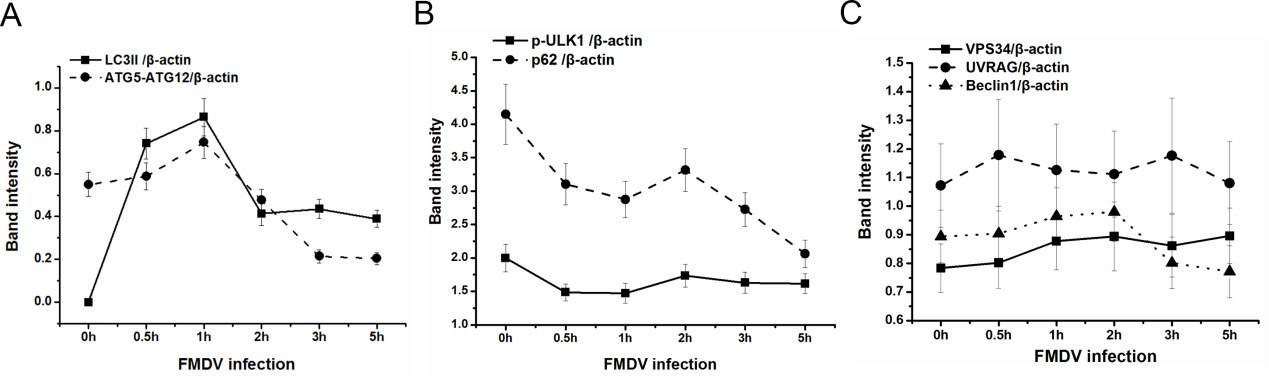
**

**Supplementary Figure 1. Quantification of western blot band density in Figure 1B.** Western blot bands of respective proteins were quantified using Image J2* software. The signals of each specific protein were normalized to the signals of β-actin. All data were analyzed using an independent sample *t*-test and expressed as the mean± standard (SD) of at least three independent samples.

**
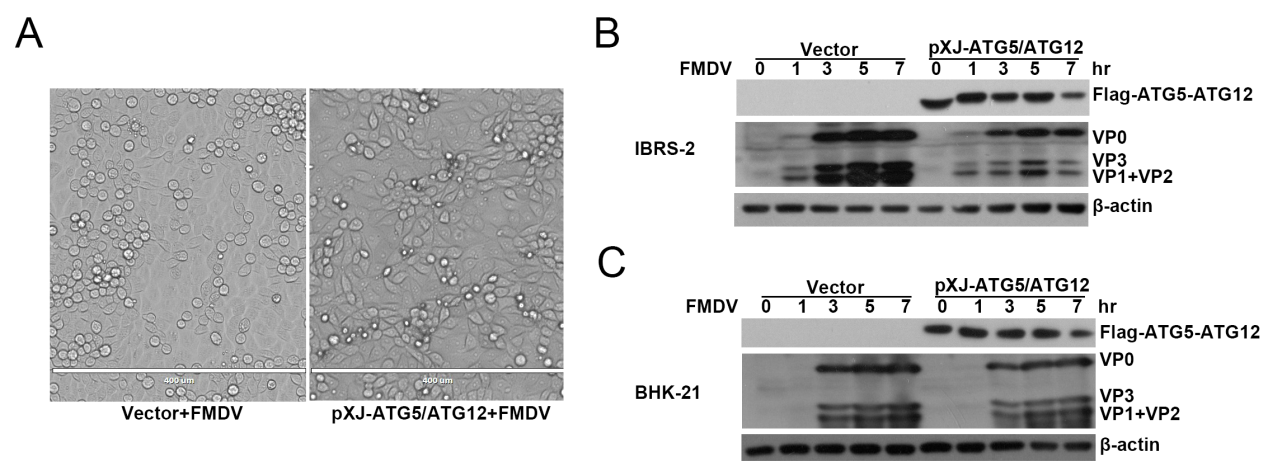
**

**Supplementary Figure 2.** (A) Overexpression of ATG5-ATG12 alleviates the cytopathic effect (CPE) upon FMDV infection. PK-15 cells were transfected with vector or Flag-ATG5 and Flag-ATG12 plasmid for 24 h, followed with FMDV infection. CPE were observed at 5 h.p.i. under ordinary optical microscope. (B) Overexpression of ATG5-ATG12 suppresses FMDV replication in IBRS-2 cells. IBRS-2 cells were transfected with vector or Flag-ATG5 and Flag-ATG12 plasmid for 24 h, followed with FMDV infection. Cells were harvested at 0, 1, 3, 5, 7 h.p.i., and the expression of Flag-ATG5-ATG12 and the level of viral proteins were analyzed with Western blot. (C) Overexpression of ATG5-ATG12 does not suppress FMDV replication in BHK-21 cells. BHK-21 cells were transfected with vector or A Flag-ATG5 and Flag-ATG12 plasmid for 24 h, followed with FMDV infection. Cells were harvested at 0, 1, 3, 5, 7 h.p.i., and the expression of Flag-ATG5-ATG12 and the level of viral proteins were analyzed with Western blot. Above experiments were
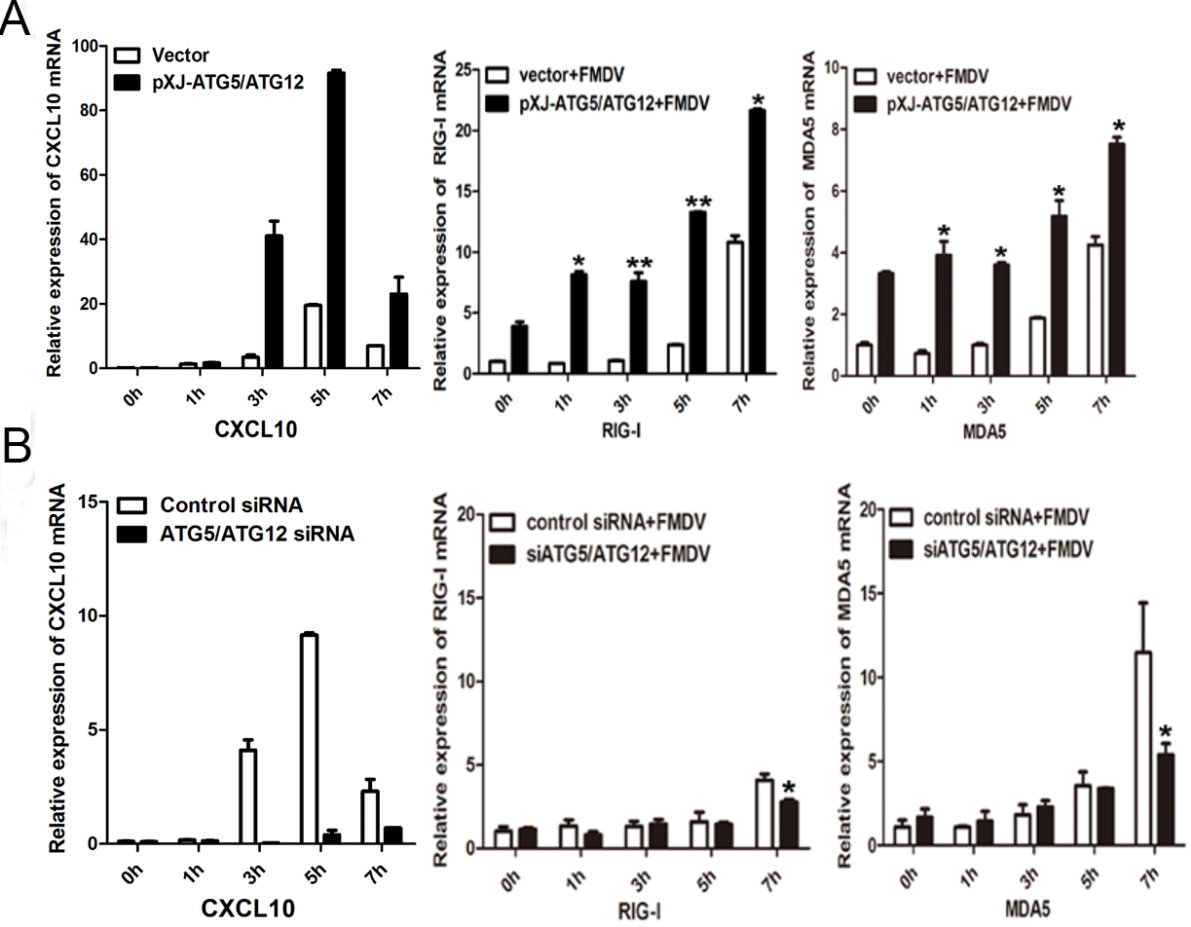
performed in triplicates.

**Supplementary Figure 3. ATG5-ATG12 promotes the expression of CXCL10, RIG-I, and MDA5.** (A) Overexpression of ATG5-ATG12 enhances the transcription of CXCL10, RIG-I and MDA5. PK-15 cells were transfected with vector, or Flag-ATG5 and Flag-ATG12 plasmid for 24 h, followed with FMDV infection. Total RNAs were extracted at 1, 1, 3, 5, 7 h.p.i., and the mRNA levels of CXCL10, RIG-I and MDA5 were quantified with real time RT-PCR. (B) Knock down of ATG5-ATG12 inhibits the transcription of CXCL10, RIG-I and MDA5. K-15 cells were transfected with control siRNA, or ATG5-ATG12 siRNA for 36 h, followed with FMDV infection. Total RNAs were extracted at 1, 1, 3, 5, 7 h.p.i., and the mRNA levels of CXCL10, RIG-I and MDA5 were quantified with real time RT-PCR. Above data are representative of three independent experiments. Graphs show mean±SD; n =3. *P＜0.05; **P＜0.01.


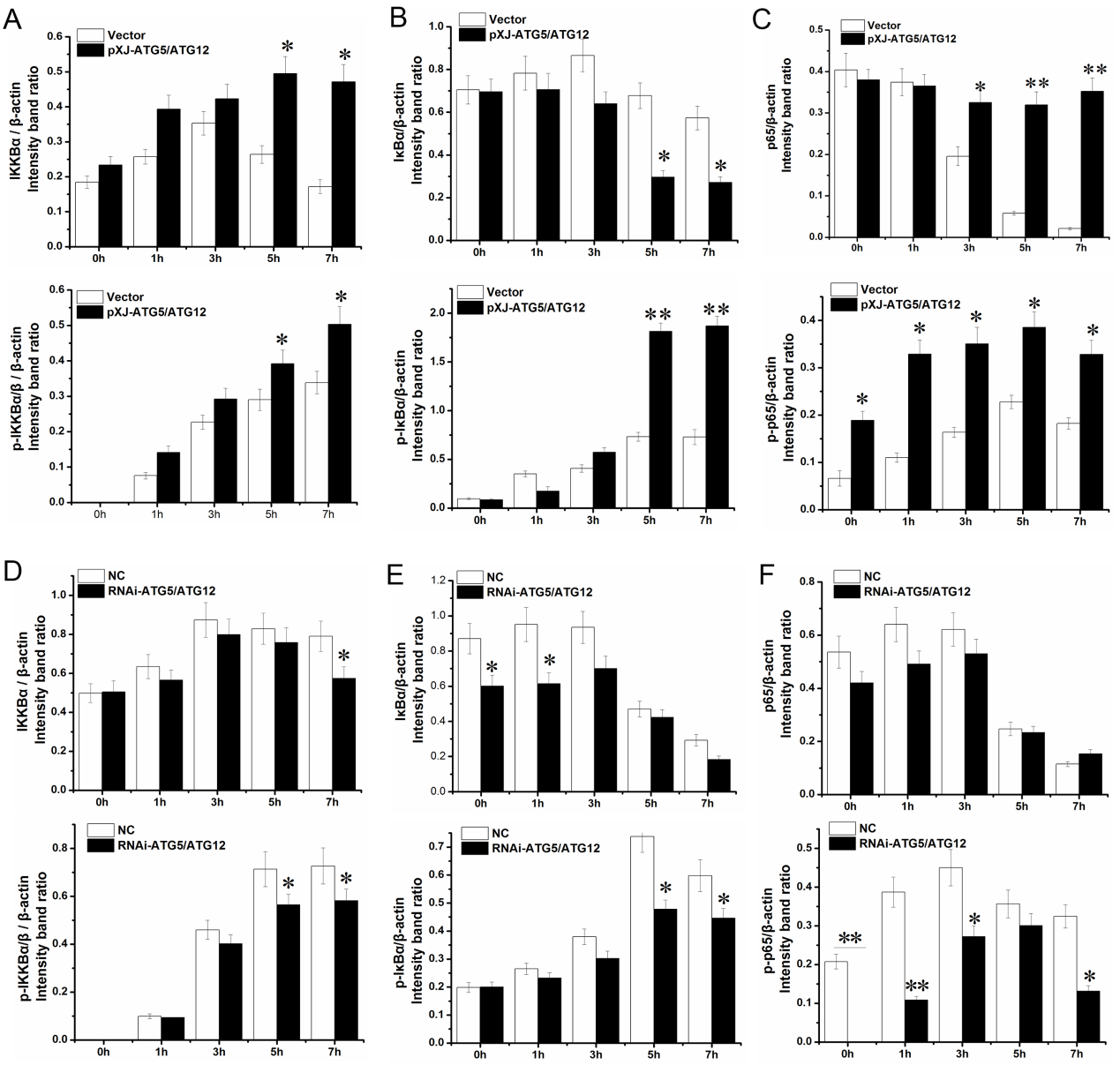


**Supplementary Figure 4.** **Quantifications of western blot band density in Figures 5A and 5B.** Western blot bands of respective proteins were quantified using Image J2* software. The signals of each specific protein were normalized to the signals of β-actin. All data were analyzed using an independent sample *t*-test and expressed as the mean± standard (SD) of at least three independent samples.


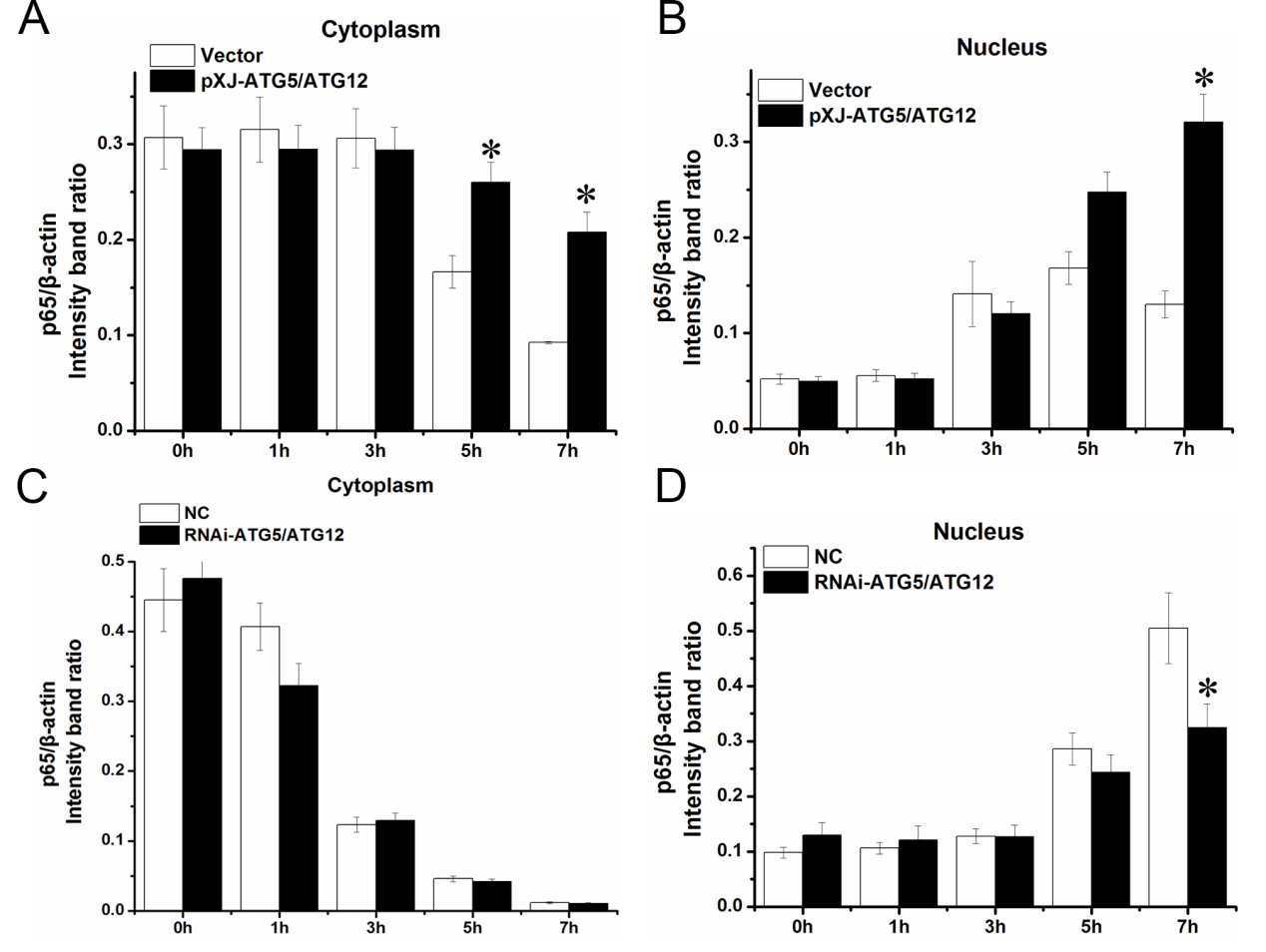


**Supplementary Figure 5. Quantifications of western blot band density in Figures 5D and 5E.** Western blot bands of respective proteins were quantified using Image J2* software. The signals of each specific protein were normalized to the signals of β-actin. All data were analyzed using an independent sample *t*-test and expressed as the mean± standard (SD) of at least three independent samples.


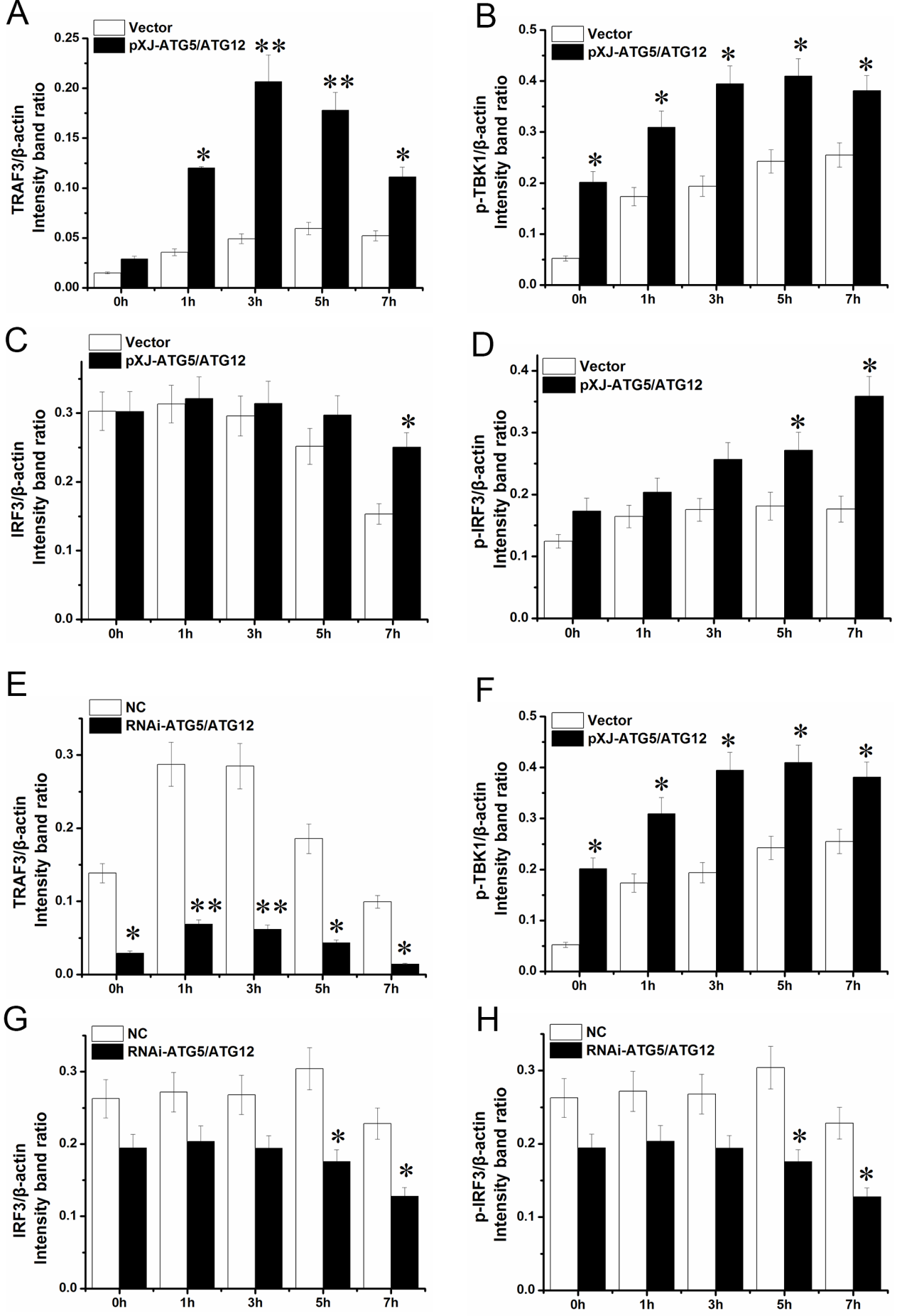


**Supplementary Figure 6.** **Quantification of western blot band density in Figure 6.** Western blot bands of respective proteins were quantified using Image J2* software. The signals of each specific protein were normalized to the signals of β-actin. All data were analyzed using an independent sample *t*-test and expressed as the mean±
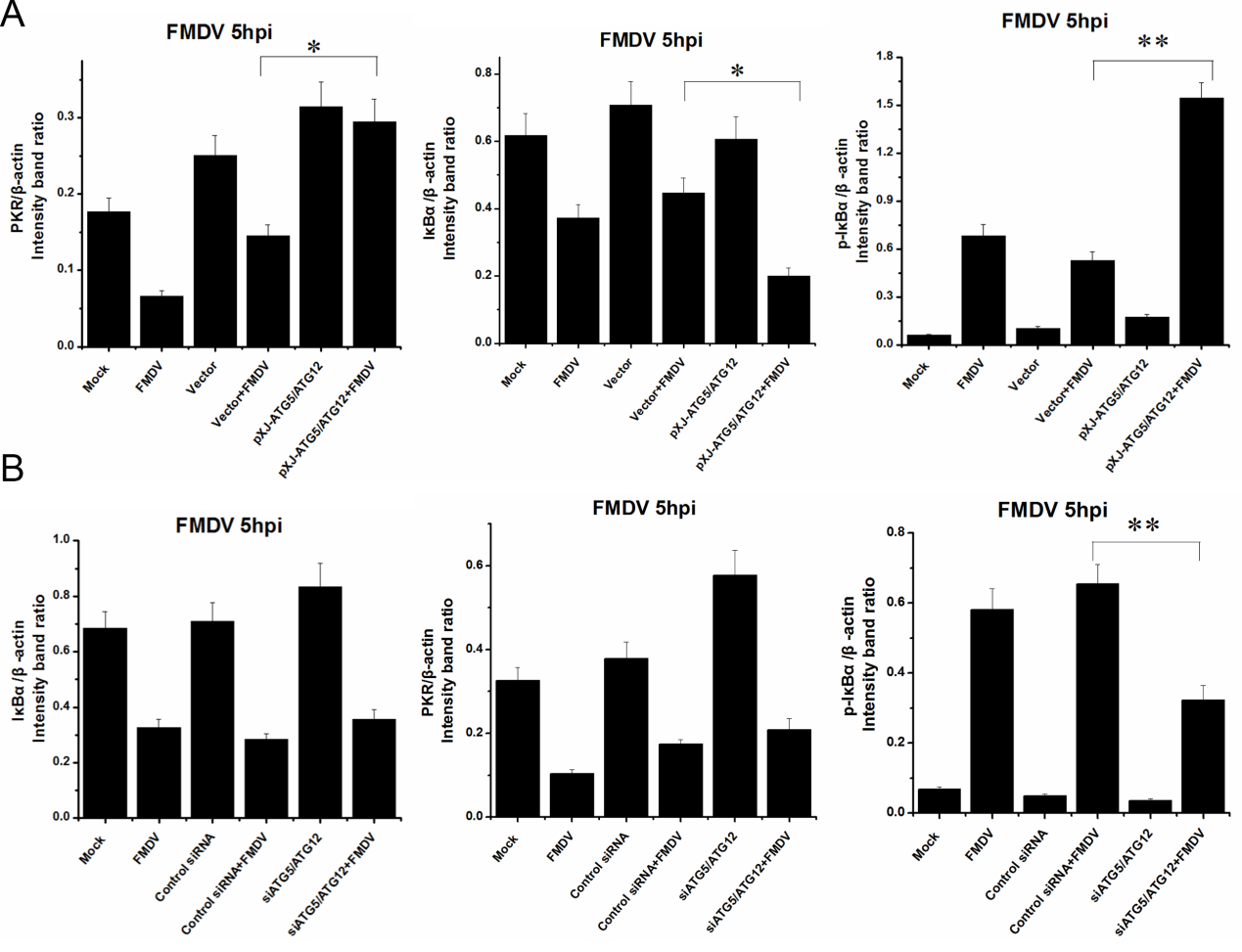
standard (SD) of at least three independent samples.

**Supplementary Figure 7 Quantifications of western blot band density in Figure 7E and 7F.** Western blot bands of respective proteins were quantified using Image J2* software. The signals of each specific protein were normalized to the signals of β-actin. All data were analyzed using an independent sample *t*-test and expressed as the mean± standard (SD) of at least three independent samples.
